# Genetic diversity and ancestry of the Khmuic-speaking ethnic groups in Thailand: a genome-wide perspective

**DOI:** 10.1038/s41598-023-43060-7

**Published:** 2023-09-21

**Authors:** Jatupol Kampuansai, Rattanasak Wongkomonched, Wibhu Kutanan, Metawee Srikummool, Tanapon Seetaraso, Suwapat Sathupak, Patcharawadee Thongkumkoon, Apiwat Sangphukieo

**Affiliations:** 1 Department of Biology, Faculty of Science, Chiang Mai University, Chiang Mai, Thailand; 2 Department of Biology, Faculty of Science, Naresuan University, Phitsanulok, Thailand; 3 Department of Biochemistry, Faculty of Medical Science, Naresuan University, Phitsanulok, Thailand; 4 Center of Multidisciplinary Technology for Advanced Medicine (CMUTEAM), Faculty of Medicine, Chiang Mai University, Chiang Mai, Thailand

**Keywords:** Evolutionary genetics, Population genetics

## Abstract

The Khmuic-speaking populations are believed to be the descendants of one of the earliest groups to settle in Mainland Southeast Asia. In Thailand, there are two agricultural Khmuic-speaking ethnic groups, the Khamu and Lua (Htin). These peoples primarily reside in scattered locations along the mountainous Thailand–Laos border in Nan province. In this study, we conducted genome-wide SNP analysis on 81 individuals from three Khamu and two Lua villages in northern Thailand. Our findings revealed that both the Khamu and Lua groups possess genetic structures that are distinct from other ethnicities in Southeast Asia, indicating a unique history of migration and settlement. Within the Khmuic group, the Khamu populations living in different locations exhibited similar genetic structures and displayed genetic affinities only with some hill-tribes and Tai-Kadai (Kra-Dai)-speaking groups in Thailand, suggesting potential intermixing or cultural exchange. Furthermore, the Lua people displayed a distinctive population structure, which could be attributed to the founder effect and endogamous marriage practices. Additionally, we discovered a relationship between the Khmuic-speaking populations in Thailand and a Neolithic ancient sample obtained from the Tham Pha Ling archaeological site in Laos. This study provides new insight into genetic substructure within the Khmuic-speaking people and their potential relationship to the indigenous inhabitants of Mainland Southeast Asia.

## Introduction

Khmuic-speaking people refers to a collection of ethnic groups who speak language that belongs to the Khmuic branch of the Austroasiatic linguistic family. They are dispersed across Laos, Vietnam, China, and Thailand. Additionally, there is a small Khamu community that was established in the United State as refugees from the Vietnam War. Although there is little known about the early history of the Khmuic-speaking people, archaeological evidence suggests that they were among the earliest peoples to arrive in Mainland Southeast Asia^1^. With a population of more than 500,000, Khmuic-speaking group make up the second-largest ethnic minority in Laos, after the majority Lao ethnicity^1^. It is generally believed that these people previously lived in a much larger area than they do now, before being pushed or absorbed by succeeding Tai-Kadai (Kra-Dai) speakers who migrated into the lowlands of Southeast Asia sometime between 1000 and 2000 years ago^2^.

In Thailand, there are two Khmuic-speaking ethnic groups, the Khamu and Lua (also known as ethnonym Htin), that are recognized by the government as hill tribes^3^. The Khamu ethnic group primarily live in the northern region of Thailand, particularly in Chiang Rai, Nan, and Phayao provinces, with a population of roughly 10,198 people. The word Khamu, and its different derivation include Kmhmu, Kemu, Khmu, and Khammu, mean “real people” or “human being”. The Khamu are thought to be one of the oldest ethnic groups of Thailand who have a long history of coexisting peacefully with their natural environment. However, many of the Khamu people in Thailand are recent immigrants from Laos and Vietnam who fled the Vietnam War and the ensuing communist regimes. Since at least 200 years ago, they have migrated over the borders to regions in Thailand for searching new agricultural land^3^. The majority of Khamu settlements are remote, however in some places, the Khamu coexist with other local minority ethnic groups.

The Lua is another hill-tribe in Thailand who speaks Khmuic language, with a population of approximately 48,025 individuals^3^. These people prefer their self-designation autonym as Lua, while the Thai government tends to call them as Htin which means local people. In some records, these people were erroneously identified as the Lawa, but they have to be distinguished from the unrelated Lawa ethnic group in northwestern Thailand. There are two subgroups of Lua people, the Mal and the Prai (or Pray), who are closely related, but have slightly difference in their spoken dialect. The majority of Lua people are dispersed throughout the mountainous region along the Thailand–Laos border, Nan province of Thailand and Sainyabuli province of Laos. There is substantial scholarly controversy over whether the Lua people had been in Thailand since prehistoric times or whether they moved from northern Laos at a later time. More likely, the Lua people began to settle in Thailand in the late 19th or early 20th century. However, some academics contend that the Lua were initially based in Nan province of Thailand before migrating to Laos and eventually returning along an old migration route to their original homeland^1^.

Marking one of the earliest genome-wide studies on the genetic diversity and structure of the Khmuic-speaking people, a Htin (Lua) population (18 individuals) from Nan province of Thailand were genotyped for roughly 50,000 autosomal SNPs along with a large geographic sample of Asian populations in 2009. The Htin (Lua) were discovered to have relatively low genetic diversity because of their small population size and isolation from other ethnic groups^4^. Further research found that these people shared recent common ancestry with the Mlabri, a group of nomadic hunter-gatherers of Thailand, and exhibited unique genetic structure that set them apart from the other ethnic groups in the country^5^.

The genetic evidence that supports the ancestral affinity between the Khamu to the Htin (Lua) and Mlabri was first reported based on mitochondrial DNA variation, and then Y-chromosome sequencing^6,7^. The same sample set of the Khmuic-speaking group, one Khamu, two Htin, and one Mlabri populations, was then reanalyzed using high resolution genome-wide SNP data. Researchers discovered that the Khmuic-speaking people shared a genetic heritage with Katuic-speaking people, another branch of the Austroasiatic language family. The study also discovered that the group of Khmuic/Katuic-speaking individuals shared their genotypes strongly within their own group but less frequently with other ethnic groups. However, the Khmuic and Katuic speaking people also showed some degree of intermarriage and cultural exchange with the Tai-Kadai-speaking groups in Laos and Northeastern Thailand that promoted their genetic affinity^8^.

Despite the fact that earlier genetic research on the Khmuic-speaking people had revealed their separate origin from other ethnic groups in the area, the small number of populations investigated prevented further insight of their diversity and ancestry. Therefore, using 81 Khmuic-speaking individuals from 5 villages in northern Thailand (three Khamu and two Lua populations), we produced an expanded genome-wide SNP data set. Allele and haplotype sharing within and between these two ethnic groups were analyzed and compared with surrounding Southeast Asian modern populations as well as ancient samples. Our findings reveal a fine-scale genetic substructure and a probable ancestry of the Khmuic-speaking people.

## Results

### Overall genetic structure of Khmuic speakers

**Figure 1:**
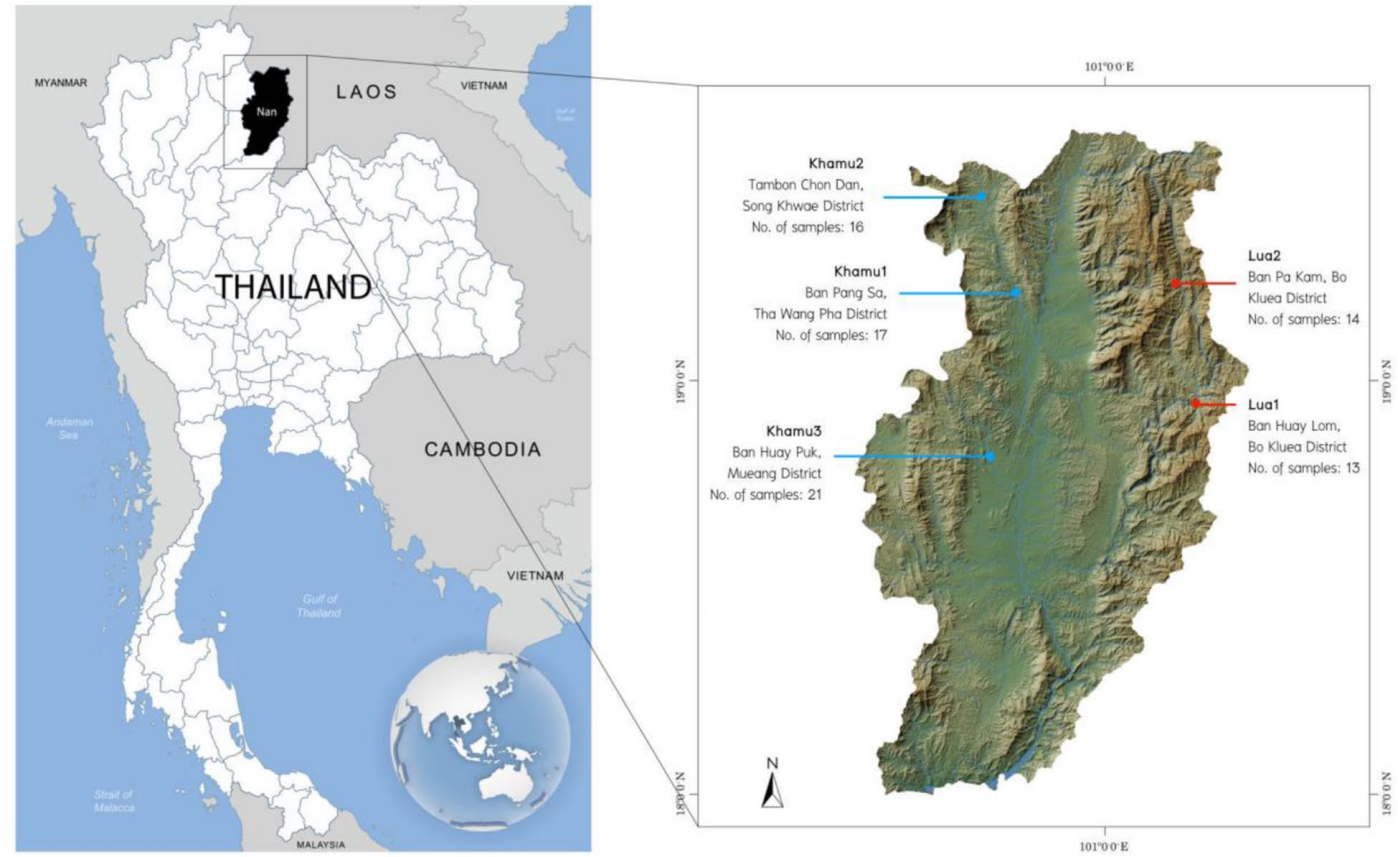
Map showing the location of the 5 Khmuic-speaking villages analyzed in this study. Drawing was adapted and used under license from Shutterstock.com. Original drawings can be found at https://www.shutterstock.com/image-vector/bluegray-detailed-map-thailand-administrative-divisions-1722049933, https://www.shutterstock.com/image-illustration/nan-province-thailand-colored-elevation-map-2281467625.

**Figure 2:**
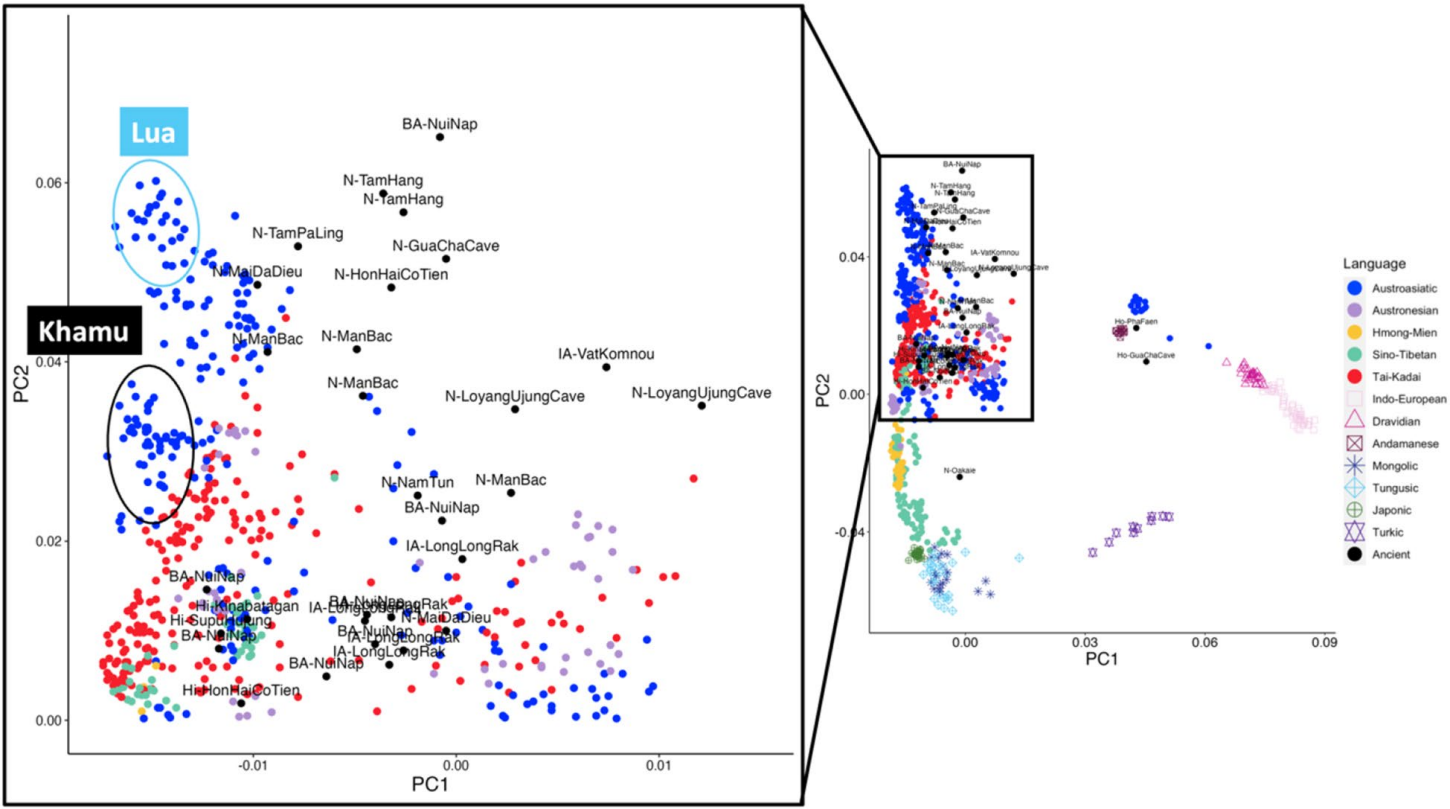
Plot of PC1 versus PC2 for the genome-wide SNP data of individuals from South Asia, Northeast Asia, and Southeast Asia is shown on the right. A high-resolution version of this plot can be found in Supplementary Fig. 1. Each individual is colored by linguistic family according to the key at the right panel. Plot focusing on the Khamu and Lua populations is zoomed-in to the left. Ancient samples are labeled for their archeological site.

We generated the genome-wide SNP data from 81 individuals belonging to the Khmuic-speaking group residing near the Thailand–Laos border in Nan province, Thailand (Fig. 1). This data was merged with a dataset of modern Asian populations generated using the same platform, as well as with ancient samples from Southeast Asia, as previously reports^8–13^. Using a set of 149,384 SNP positions, we analyzed a total of 959 individuals, including various Asian samples, and plotted the results on a PCA plot (Fig. 2, Supplementary Fig. 1). The plot revealed distinct separation between East Asian populations on the left and South Asian populations on the right along the PC1. Along the PC2 axis, the East Asian populations further divided into two distinct clusters: Northeast Asians in the lower-left and Southeast Asians in the upper-left. When considering language families, the Sino-Tibetan and Hmong-Mien speaking populations in the lower-left exhibited notable genetic differences from the Austroasiatic, Tai-Kadai, and Austronesian-speaking populations in the upper-left. We observed that our newly studied groups, the Khamu and Lua populations, clustered together with the Htin populations from previous studies. These groups displayed genetic distinctions from other ethnic groups within Thailand and other East Asian populations. Additionally, a group of Khmuic-speaking individuals exhibited genetic affinities with ancient DNA data from the Neolithic period in some regions such as N-Mai Da Dieu and N-Man Bac in Vietnam, and N-Tam Pa Ling in Laos.

**Figure 3:**
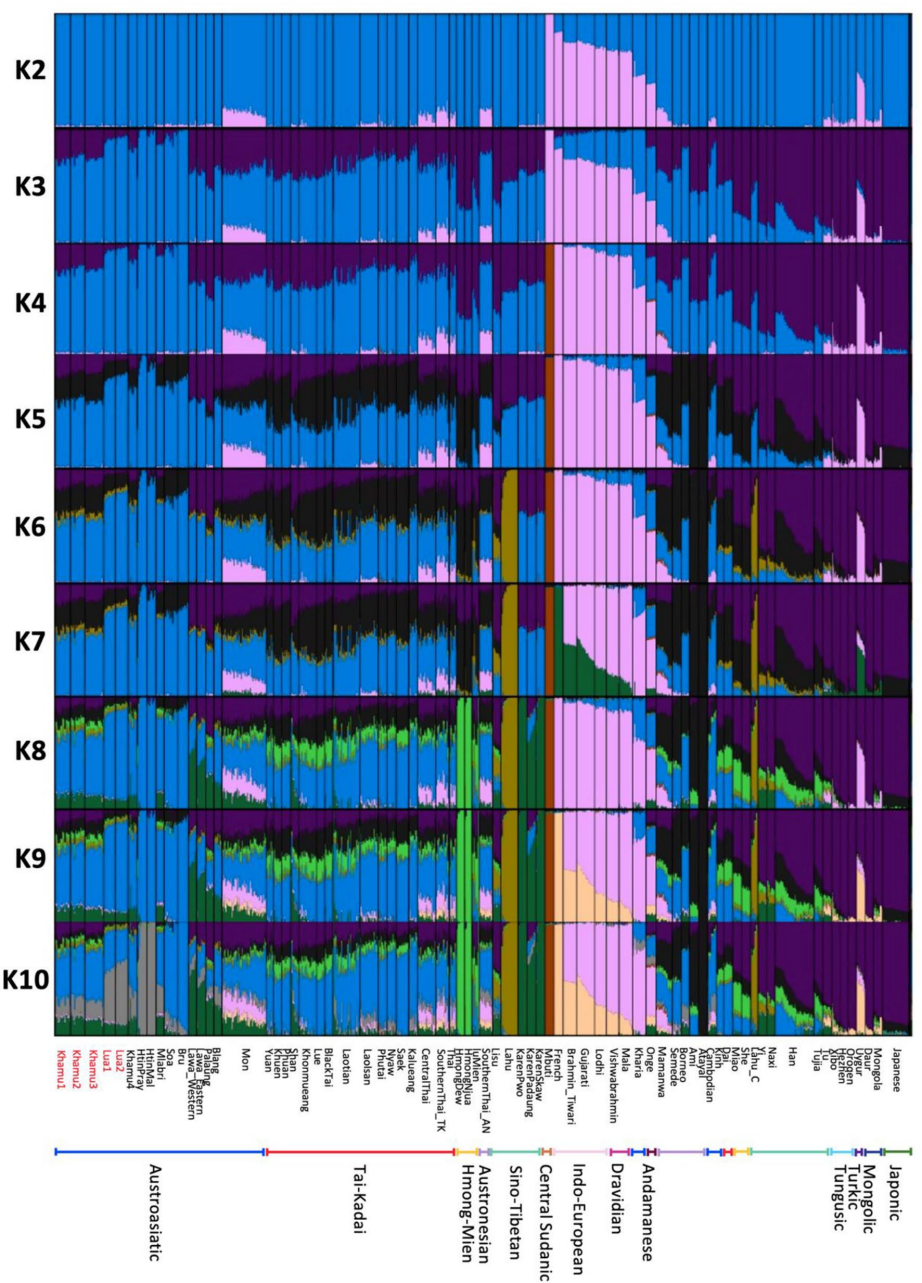
ADMIXTURE results of modern Asian populations and the outgroup, African Mbuti and European French, for K values ranging from 2 to 10. Each individual is represented by a bar divided into K colored segments, indicating their estimated membership fractions in each of the K ancestry component. Populations are separated by black lines. The names of the populations whose genome-wide data are generated in this study are indicated in red on the lower-left side. The bar at the bottom represents the linguistic families of each ethnic group.

We used the ADMIXTURE version 1.3.0 program^14^ to investigate the sources of population structure within each population. A total of 155,709 SNP positions from a set of 979 individuals were involved in this analysis, encompassing Asian samples along with the African Mbuti and European French populations serving as the outgroup. We divided the gene pool into K groups, ranging from 2 to 10 groups, and performed 100 repetitions for each grouping. We projected ancient DNA samples and populations that exhibited significant genetic differences from other populations, including Onge, Mamanwa, Mlabri, Khamu, and Lua. The analysis revealed that the cross-validation value was lowest at K = 4 (Supplementary Fig. 2), indicating that the studied populations are best divided into four groups. At K = 4, we observed distinct genetic components in different populations. The Mbuti population exhibited a unique and separate genetic component represented by a dark brown color. The pink component was prevalent in the French population and Indian groups (Dravidian/Indo-European-speakers). The purple component constituted a major proportion of populations from Northeast Asia. Among the samples from Thailand who speak Austroasiatic and Tai-Kadai languages, the two primary components were blue and purple (Fig. 3). In the Khamu and Lua ethnic groups of our study, we found that their genetic structures were similar to other populations in Thailand, characterized by two main components, blue and purple. However, the Khamu population had a higher proportion of the purple component compared to the Lua population. Interestingly, even with increasing K values, both the Khamu and Lua populations maintained a significant proportion of both genetic components. When we reached K = 10, although higher K values were associated with increased cross-validation errors, specific components unique to the Khmuic language group (Khamu, Lua, Htin), represented by grey in Fig. 3, were predominantly found within this group and in the ancient DNA samples from Laos and Vietnam (Supplementary Fig. 3).

### Allelle sharing and genetics affinity

**Figure 4:**
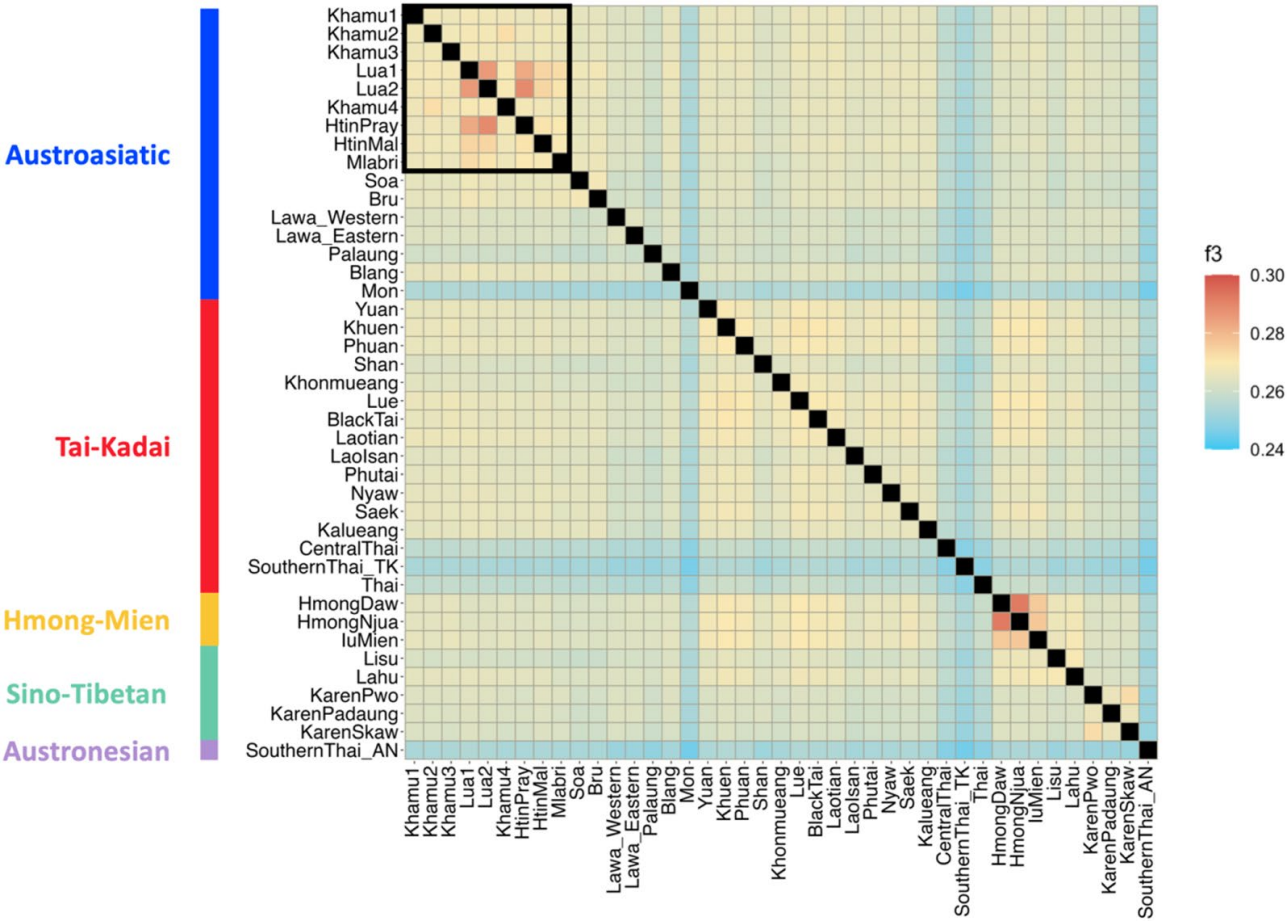
Heatmap showing population allele sharing profiles based on *f3* statistics. The colored bar on the right map indicates the statistical values, while that on the left side indicates the linguistic family of each ethnic group. The Khmuic-speaking populations are emphasized with a black box in the upper-left corner.

To examine population relationships based on allele sharing, we calculated outgroup *f3*-statistics in the form *f3*(X, Y; outgroup) to measure genetic affinity between populations X and Y when separated from the outgroup (Mbuti population). Higher *f3*-statistics indicate closer genetic affinity between populations. Among various ethnicities in Thailand, the Thai and Mon populations had the lowest *f3*-statistics compared to other ethnic groups, including ancient DNA. Conversely, the Lua, Htin, and Hmong populations exhibited the highest *f3*-statistics. Notably, the Lua population displayed close genetic affinity with the Htin population, while the Khamu populations exhibited the greatest genetic diversity within the Khmuic language group (Fig. 4, Supplementary Table 3). When analyzing the allele sharing with ancient samples, the Lua populations showed the closest genetic affinity with Neolithic DNA from Tam Pa Ling and Tam Hang in Laos (Supplementary Fig. 4). The haplotype sharing profiles as inferred by the ChromoPainter analyses also confirm that the Khmuic-speaking people exhibit close relationship within their cluster (Supplementary Fig. 5).

**Figure 5:**
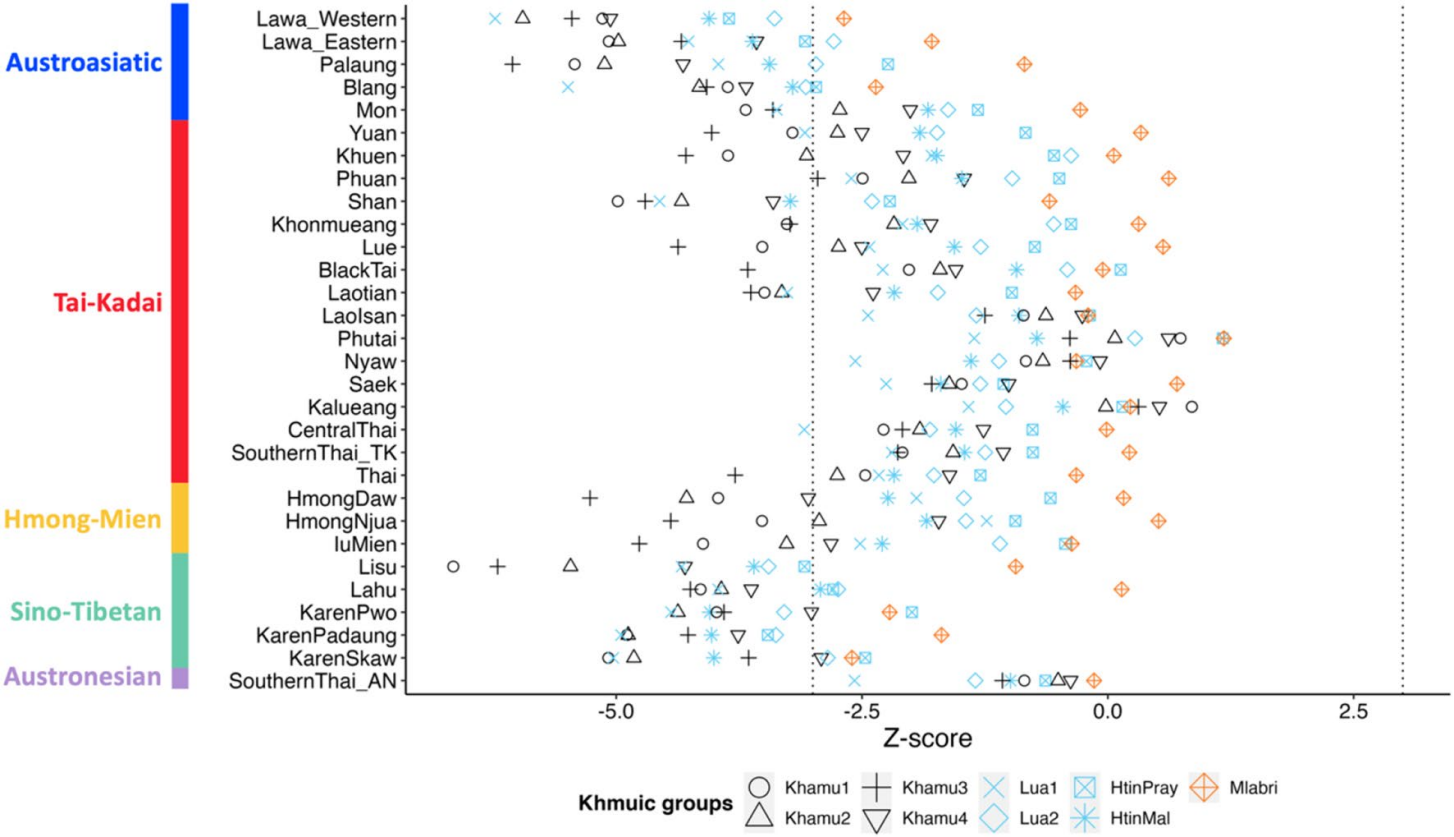
*f4* statistics comparing Khmuic-speaking populations from two different subgroups of the Austroasiatic language family. Z-scores are for *f4* (W, X; Y, Mbuti), where W is a selected Austroasiatic-speaking Soa ethnic group, X is another population in Khmuic branch in Austroasiatic family, and Y is a population not in Austroasiatic family whose ethnic name was labeled on the left side. Different symbols denote different populations for X: black corresponds to Khamu, blue to Lua/Htin, and orange to Mlabri. The bar on the left represents the linguistic family of each ethnic group. The vertical black dashed lines denote + 3/− 3.

**Figure 6:**
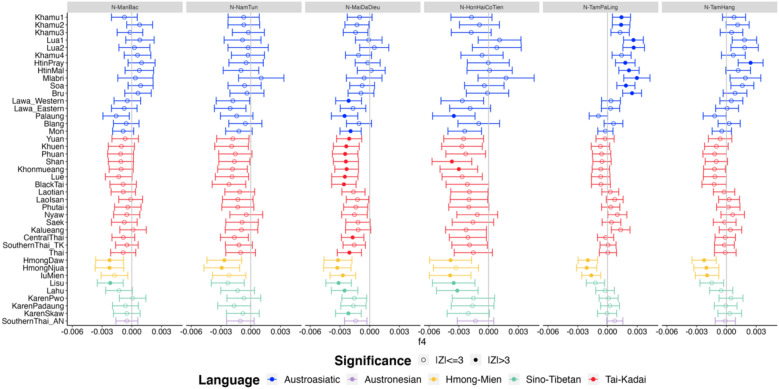
*f4* statistics comparing ethnic populations in Thailand labeled on the left to Neolithic ancient samples from Laos and Vietnam on the top grey bar. Z-scores are for *f4* (ancient sample, Han Chinese; ethnic population, French). The vertical grey lines denote 0. The dots and error bars are colored according to language family, as indicated by the key at the bottom. Empty circles denote nonsignificant Z-scores (|Z|= < 3) and solid circles denote significant Z-scores (|Z|> 3).

Next, we investigated population relationships using *f4*-statistics in the format of *f4* (W, X; Y, Mbuti), where W represents a selected Austroasiatic-speaking Soa ethnic group, X is another population in the Khmuic branch of the Austroasiatic family, and Y is a population outside the Austroasiatic family. Conventionally, a Z-score > 3 or <  − 3 indicates that Y shares significant excess ancestry with W or X, respectively. Nonsignificant Z-scores indicate that W and X form a clade and share equivalent amounts of ancestry with Y. Please take note that the Austroasiatic-speaking Bru population, which exhibits similar genetic structure to our chosen reference group, the Soa population, has not been investigated in this analysis. According to the *f4*-statistics, we found that the Khmuic-speaking populations closely aligned with the groups speaking Austroasiatic languages, particularly the Palaungic branch (Lawa, Palaung, and Blang). When comparing the Khamu and Lua populations, the Khamu exhibited closer genetic affinity with the Tai-Kadai language group and some hill tribes of Thailand, such as Hmong, IuMien, and Lisu (Fig. 5, Supplementary Table 4 and 5). We further investigated the groupings of modern populations and ancient samples by *f4*-statistics. By employing the form *f4* (ancient sample, Han Chinese; ethnic population, French), we assessed if any ethnic group in Thailand displayed affinity with Southeast Asian ancient samples compared to the Han Chinese (Supplementary Fig. 6). Most of the Khmuic-speaking people exhibited the closest genetic affinity with ancient DNA from Tam Pa Ling in Laos, especially the Lua and Mlabri populations who show high significance (Fig. 6).

### Genetic ancestry of the Khmuic-speaking people

**Figure 7:**
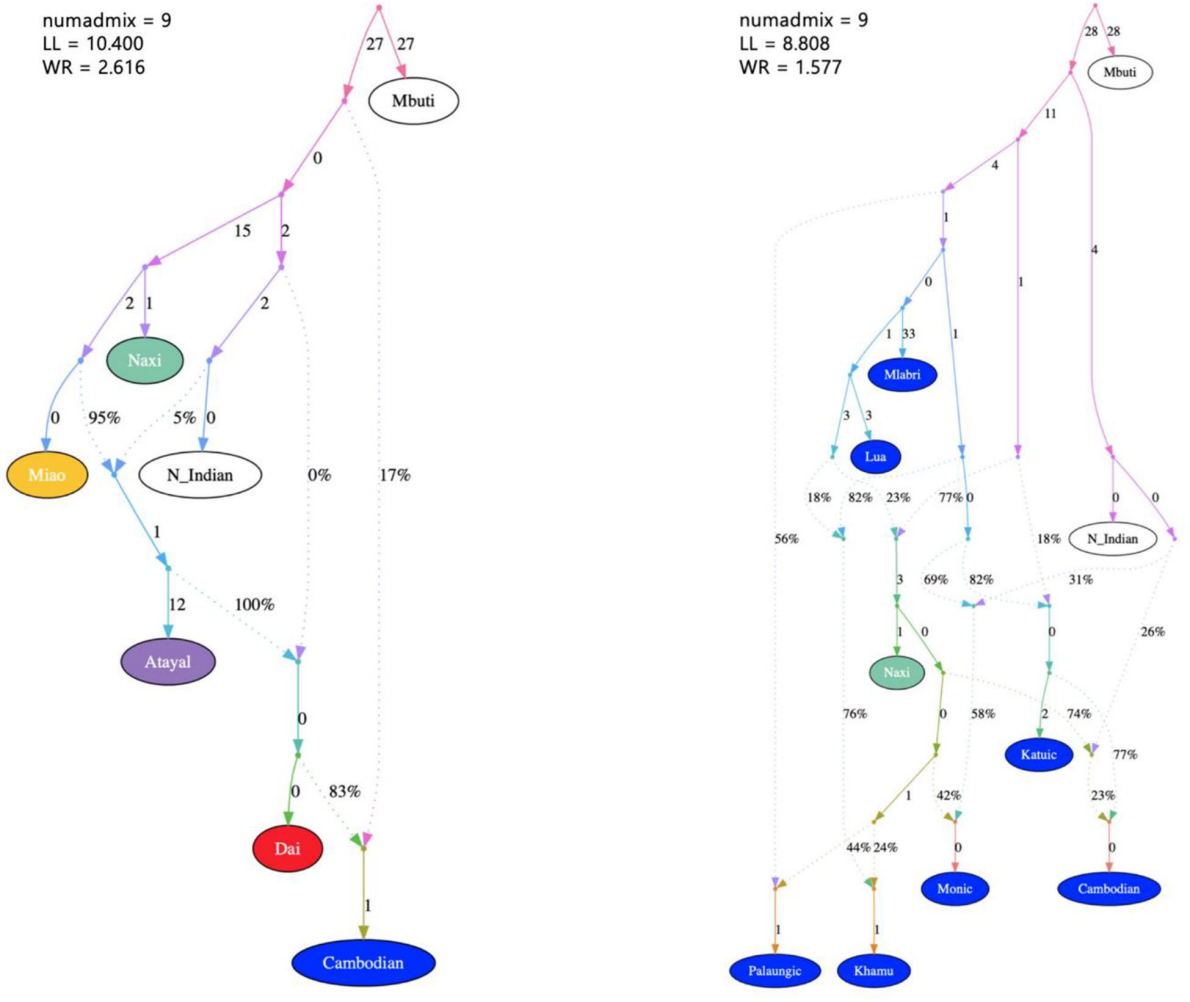
Admixture graphs for the backbone population (left) and the Austroasiatic-speaking group (right). Backbone population labels are colored for different language family. Dashed arrows represent admixture edges, while solid arrows are drift edges reported in units of Fst × 1000.

Finally, we constructed admixture graphs to determine the ancestry of the Khmuic-speaking people. The Mbuti and North Indian populations were served as outgroups, while we selected representative ethnic groups from each linguistic family using the *f4*-statistic test (Supplementary Fig. 7). The Atayal, Dai, Cambodian, Miao, and Naxi were chosen as representatives of the Austronesian, Tai-Kadai, Austroasiatic, Hmong-Mien, and Sino-Tibetan language families, respectively. In the backbone graph, the initial split separates the North Indian- and Naxi-related ancestries. The Miao lineage is closely related to the Naxi-related ancestry. The ancestors of Atayal and Dai are a mixture of North Indian- and Naxi/Miao-related ancestries. The Cambodian ancestor is a mix of 83% from the Dai ancestry and 17% from the ancestry of all East Asian groups (Fig. 7).

Next, we included the Khamu and Lua populations into the admixture graph, along with other groups from the Austroasiatic language family, including the Mlabri, Cambodian, Paluangic (Lawa, Paluang, Blang), Monic (Mon), and Katuic (Soa, Bru) (Fig. 7). After separating the outgroup, we observed that the Mlabri and Lua populations are among the earlier group to differentiate from the others. The Palaungic ancestry is the result of a mixture between the ancestral populations of all Austroasiatic groups and the Naxi-related ancestry in a ratio of 56% and 44%, respectively. The Khamu ethnic group appears to be descendants of the Mlabri/Lua lineage (76%) with some intermixing of the Naxi-related ancestry (24%). The Monic, Katuic, and Cambodian populations inherited varying levels of North Indian ancestry, which distinguishes them from the Paluangic and Khmuic language groups (Fig. 7).

## Discussion

Ethnic groups of Thailand that speaks language belonging to the Khmuic branch of the Austroasiatic linguistic family, consists of two major ethnicities, the Khamu and Lua (or ethnonym as Htin). These groups have settled along the Thailand–Laos border, particularly in Nan province^3^. Through the analysis of high-resolution autosomal markers, we discovered that the genetic structure of the Khamu and Lua people differs significantly from other ethnic groups in Thailand and East Asia. The outgroup *f3*-statistics have indicated a significant degree of genetic drift shared among populations speaking the Khmuic language (Fig. 4). While our analyses do not allow for definitive conclusions regarding the exact evolutionary forces driving the genetic distinctiveness of these Khmuic-speaking people, it appears that factors such as founder effects and geographical isolation have played pivotal roles in shaping their genetic profile. The migratory history of the Khamu and Lua communities in Laos and Thailand is marked by numerous movements and instances of fragmentation^1,3^. Previous research on uniparental markers, including approximately 2.34 mB of the Y-chromosome and the complete mitochondrial genome, has revealed that the Khmuic-speaking Htin (Lua) ethnic group demonstrates reduced diversity and their effective population size decreasing around 2000–2500 years ago, especially subsequent to the migration of Tai-Kadai-speaking populations into Southeast Asia roughly 1000–2000 years ago^15^. This observation suggests that the genetic composition of contemporary Khmuic-speaking populations could have been notably influenced by a restricted number of initial settlers, resulting in decreased genetic diversity and heightened differentiation from other groups. However, a more comprehensive exploration involving various uniparentally inherited markers within the Khamu and Lua populations is required to elucidate the precise impact of the founder effect on these ethnic groups. Additionally, the mountainous northern region of Nan Province, where the Khamu and Lua villages are located (Fig. 1), has limited communication with other populations, contributing to the preservation of their unique cultural structure over time. Factors such as linguistic differences from the majority Thai population and the practice of endogamous marriage within the Khamu and Lua communities further contribute to their distinct genetic structure, setting them apart from other ethnic groups.

Within the Khmuic-speaking group, a comparison of different Khamu populations living in various areas of Nan province, such as Mueang District, Tha Wang Pha District, and Song Khwae District (Fig. 1), revealed similar genetic structures. Interestingly, the Khamu show closer relationships with other ethnic groups compared to the Lua, particularly with the Tai-Kadai-speaking groups like Tai Lue, Tai Yuan, and Tai Khuen (Fig. 2). This is consistent with the historical background of the Khamu people who migrated to Thailand and became involved in elephant handling, logging, and lumberjack activities for French timber companies in the nineteenth century AD^1^. Some Khamu individuals settled in Thailand and interacted with lowland Tai-Kadai-speaking groups, resulting in cultural exchanges and possible intermarriage. For example, some Khamu people wear hand-woven textiles similar to those of the Tai Lue and are referred to as “Khamu Lue” reflecting the cultural influence of the Tai Lue in Khamu communities.

Furthermore, our genome-wide analysis indicates a relationship between the Khamu and some hill-tribe groups such as Hmong, IuMien, and Lisu (Fig. 5). In the past, intermarriage between different hill-tribe groups was rare due to differences in traditions and localities. However, in the modern world, the genetic and cultural boundaries among these groups are gradually easing, leading to increase their genetic affinity. The inter-ethnic marriage between the Khmuic- and the Hmong-Mien-speaking people who live in neighboring area both in Thailand and Laos is presently possible. It is worth noting that some anthropologists believe that the Khamu originally migrated southwards from northern Myanmar and the southwestern part of Yunnan province in China^1^, where Tibeto-Burman-speaking people of the Sino-Tibetan language group are prevalent. The presence of Naxi-related ancestry in the Khamu of Thailand gene pool as seen in our result (Fig. 7), suggests a shared common ancestor in the past, but further investigation is needed to clarify this genetic connection.

We found that the genetic structure of the Lua people is unique and different from other ethnic groups in Mainland Southeast Asia. However, the Lua people are genetic closely related to the previously identified Htin group. It is worth mentioning that the terms “Lua” and “Htin” are used interchangeably by the government, with residents referring to themselves as the “Lua” and the term “Htin” being employed by government officials to denote them as indigenous inhabitants (Htin means locality in Thai language). Our genome-wide genotyping confirms that these two ethnic groups are, in fact, the same group. Despite the Lua people increasingly engage into lowland day-labor and their lifestyle gradually blend with other ethnic groups, our comprehensive genomic data still demonstrates that the genetic structure of the Lua people in Nan province remains distinct and different from that of other ethnic groups. This finding is consistent to a previous study based on mitochondrial DNA analysis, which identified unique haplogroups B6a, F1a1a, and M12a1a exclusive to the Lua (Htin) people and absent in any other ethnic groups in the northern region of Thailand^16^. The distinctive genetic structure observed in the Lua population challenges the assumption of a close relationship between two branches of the Austroasiatic family, the Khmuic and Katuic, as previously observed based on genome-wide autosomal markers^8^. While certain Khmuic-speaking ethnic groups, like the Mlabri and HtinMal, display a notable relationship with Katuic speakers, the Lua population appears to share genetic drift primarily within their Khmuic branch rather than with the Katuic-speakers (Fig. 4, Supplementary Fig. 5). Our admixture graph results also corroborate this, indicating that the Lua population possesses a distinct genetic history and maintains differentiation from the Katuic-speaking group (Fig. 7).

There is a small group of hunting-gathering nomads living in northern Thailand, the Mlabri. The term “Mlabri” translates to “people in the forest,” combining “Mla” meaning people and “Bri” meaning forest. They have a distinct characterized by a nomadic lifestyle, constantly moving without permanent settlements. Some scholars propose that the Mlabri people are direct descendants of the Hoabinhian group, which was the original population in Southeast Asia^17^. Currently, the Mlabri people are found only in two provinces of Thailand, namely Phrae and Nan, with a population of approximately 400 individuals^18^. Previous studies using genomic analysis have supported genetic affinities between the Mlabri and the HtinMal groups^5^. Furthermore, uniparental markers indicate paternal relationships among the Mlabri, HtinMal, HtinPray, and Khamu ethnic groups, while maternal genetic relationships are observed between the Mlabri and the Soa and Bru groups, who speak the Katuic language in northeastern Thailand^7^. Our present genomic data also confirm the close relationship between the Khmuic-speaking ethnic groups and the Mlabri, with the Lua people being closer to the Mlabri than the Khamu (Fig. 4, 7). This finding is consistent with linguistic evidence that groups these ethnicities together in the Austroasiatic language family, specifically within the Khmuic branch. Linguist used a technique of comparing changes in pronunciation of words and proposed that the Lua (Htin) people separated from the Khamu ethnic group about 600 years ago and then separated into two groups, the Mal and the Pray, about 200–300 years ago. However, linguistic data cannot definitively indicate when the Lua and the Mlabri people separated from each other, but it is estimated to have been several hundred years ago^19^. Both the genetic and linguistic evidence suggest that the Khamu and Lua (Htin) of Khmuic language group share the same ancestry with the Mlabri, who is believed to be the descendants of indigenous inhabitants of Mainland Southeast Asia.

Comparisons of genomic data from various ethnicities in Southeast Asia with ancient DNA have revealed intriguing connections between the Khmuic language group and ancient DNA from Tam Pa Ling site in Laos, near the border to Vietnam. This archaeological site has yielded human remains dating back at least 60,000 years, challenging previous assumptions that humans migrated along coastlines out of Africa. Instead, it is possible that human migration may have followed natural river valleys through the continent. The ancient DNA data from Tham Pha Ling and Tham Hang caves were studied and reported in 2018^9^. The DNA was extracted from human remains aged approximately 2378–3071 years ago represents a link between the end of the Neolithic period and the Bronze Age. The genetic relationship between the Khmuic language group and the ancient DNA from Laos (Fig. 6), as evidenced by the discovery in Tham Pha Ling site, sheds light on the continuous development of the people in northern Laos from ancient times to the present. While direct historical connections between the Khmuic-speaking people in Thailand and neighboring countries remain absent, except for Laos, preceding genetic studies have illuminated the influence of these prehistoric inhabitants on various Asian populations. A genome-wide autosomal study involving approximately 55,000 SNPs has elucidated the genetic element of Khmuic-related ancestry (as exemplified by Htin and Mlabri) within the Kinh, the predominant ethnic group in Vietnam. The admixture of Chinese and Southeast Asian lineages, particularly the Proto-Malay and Khmuic residences, has likely played a significant role in shaping the present-day Vietnamese population^20^. The genetic component of Khmuic speakers (HtinMal and Mlabri) predominantly reflects a first neolithic farmer-like source, a conclusion supported by comprehensive genome-wide SNP data. This Khmuic ancestry, alongside the Neolithic Fujianese-like source prevalent among Austronesian speakers, contributes to the preservation of genetic sub-structure within Southern China, a phenomenon established prior to the Holocene^21^. The presence of Khmuic ancestry across various Southeast Asian populations underscores the importance of investigating genetic information of these people. Our admixture graph results (Fig. 7) reveal that some Khmuic-speaking groups in Thailand, specifically the Lua and Mlabri, were among the earliest to diverge from other ethnic groups in the region, possibly without significant genetic admixture. Thus, these ethnic groups hold paramount significance as prehistoric descendants in the study of human evolution in Mainland Southeast Asia.

However, like many ethnic minorities in Thailand, the Khmuic-speaking people have faced challenges of admixture and cultural assimilation. Many Khmuic individuals have been encouraged to adopt Thai language and culture to assimilate into mainstream Thai society. This has led to a loss of traditional knowledge and practices, with younger generations less likely to speak the Khmuic language or engage in traditional beliefs and rituals. Conducting further studies with larger sample sizes and more comprehensive genetic markers to explore specific lineages and establish genetic links between this group and other ancient samples may not be feasible in the near future. Hence, there is an urgent need to expedite genetic and cultural research among these prehistoric descendants and their relatives in neighboring countries, especially in Laos where majority Khmuic people reside, to gain a comprehensive understanding of the human evolution knowledge in Southeast Asia. Although our result prompts an overview of the genetic structure and ancestry of the Khmuic-speaking ethnic groups in Thailand, we wish to emphasize that our methodologies were rooted in the neutral theory, whose assumptions are pertinent only to a limited scope of genetic diversity^22,23^. Our conclusions remain provisional, and forthcoming theories of molecular evolution have the potential to offer a progressively broader approach to exploring the diversity and ancestry of the Khmuic-speaking people.

## Conclusion

We generated and analyzed an extensive genome-wide SNP data set from 81 Khmuic-speaking people from 5 villages (3 Khamu and 2 Lua) living in northern Thailand. Our findings revealed that both the Khamu and Lua ethnic groups possess genetic structures that are distinct from other ethnicities in Thailand and Southeast Asia, indicating a unique history of migration and settlement. Upon examining within the Khmuic linguistic group, we observed that the Khamu populations living in different locations displayed similar genetic structures. Moreover, they exhibited genetic affinities with some hill-tribes and Tai-Kadai-speaking groups in Thailand, suggesting possible intermixing or cultural exchange over time. On the other hand, the Lua people displayed a distinct population structure, likely influenced by the founder effect and endogamous marriage practices.

A significant finding of this study was the discovery of a genetic relationship between the Khmuic-speaking people in Thailand, particularly the Lua subgroup, and the Neolithic ancient DNA obtained from the archaeological site in Laos. This close genetic relationship between ancient DNA and the present-day ethnic group highlights the continuity of the population's development from ancient times to the present. This study provides new insight into genetic substructure within the Khmuic-speaking people and their potential relationship to the indigenous inhabitants of Mainland Southeast Asia.

## Methods

### Ethical statement

Ethical approval of this study was granted from the Human Experimentation Committee of the Research Institute for Health Sciences, Chiang Mai university, Thailand (Certificate of Ethical Clearance No. 31/2022). During the research, we protect the rights of participants and their identity, and we confirm that all experiments were performed in accordance with relevant guidelines and regulations based on the experimental protocol on human subjects under the Declaration of Helsinki. Written informed consent from all volunteers was obtained prior to the interview and sample collection.

### Sample collection and quality control

A total of 95 unrelated subjects residing in five villages of Nan province, Thailand, were enrolled with written informed consent. Volunteers were healthy subjects who were over 20 years old, of Khmuic-speaking ethnicity and had no ancestors that were known to be from other recognized ethnic groups for at least three generations. We collected personal data using form-based oral interviews for self-reported unrelated lineages, linguistics, and migration histories. Following the manufacturer's instructions, we collected buccal or saliva samples and extracted DNA using the Gentra Puregene Buccal Cell Kit (Qiagen, Germany).

Genotyping was carried out using the Affymetrix Axiom Genome-Wide Human Origins array^10^. Affymetrix Genotyping Console v4.2’s primary screening produced a total of 93 samples that were genotyped for 622,834 loci on the hg19 version of the human reference genome coordinates (genotype call rate ≥ 97%). We used PLINK version 1.90b5.2^24^ to exclude loci and individuals with more than 5% missing data and also exclude mtDNA and sex chromosome from our analysis. We further excluded loci that did not pass the Hardy–Weinberg equilibrium test (*P* value < 0.00005) or had more than 5% missing data, within any population. We used KING 2.3^25^ to determine individual relatedness, and we removed one person from each pair of first degree kinship. After these quality control measures, there are 81 Khmuic-speaking people (Fig. 1) with 612,614 loci overall.

We next used PLINK version 1.90b5.2 to merge our newly obtained genotyping results with a set of genome-wide SNP data^8^, which included populations from East/Southeast Asia, South Asia, African Mbuti, European French, and Southeast Asian ancient samples^9–13^. It should be noted that in this collection, allelic data from ancient samples was gathered using pseudo-haploid techniques, and samples with less than 15,000 informative loci were eliminated. After filtering the positions of SNPs that can be jointly analyzed within this dataset, we excluded SNPs that had more than 5% missing data or with a minor allele frequency (MAF) less than 3.3×10^–4^ or were not in Hardy–Weinberg equilibrium with a significance level of *P* < 0.00005. As a result, 353,505 positions in a dataset consisting of 979 individuals from 90 populations (Supplementary Table 1 and 2) were used for subsequent analysis.

### Population structure analyses

In order to investigate the genetic structure and relationships of the analyzed sample, we used PLINK version 1.90b5.2 to perform pruning for linkage disequilibrium, excluding one variant from pairs with *r*^2^ > 0.4 within windows of 200 variants and a step size of 25 variants. A total of 959 individuals from the sample set, excluding the Mbuti and French populations, were incorporated. There were 149,384 SNPs positions available for this analysis. The Principal Component Analysis (PCA) was carried out using smartpca from EIGENSOFT with the "lsqproject" and "autoshrink" options.

To infer population structure, we employed 155,709 SNP positions derived from a sample set of 979 individuals, which encompassed both Asian samples and the outgroups represented by the Mbuti and French populations, for the ADMIXTURE analysis. The clustering tool ADMIXTURE version 1.3.0^14^ was run from K = 2 to K = 10 with 100 replicates for each K and using random seeds with the -P option. For each *K*, the top 20 ADMIXTURE replicates with the highest likelihood for the major mode were displayed using PONG version 1.4.7^26^. For these PCA and ADMIXTURE analyses, the ancient samples and highly drifted modern populations (Mlabri, Onge, Mamanwa, Khamu, and Lua) were projected.

### Allele and haplotype sharing analyses

To test admixture and excess ancestry sharing, we used admixr version 0.7.1^27^ from ADMIXTOOLS version 5.1^10^ to calculate the *f3* and *f4*-statistics, with assessed through block jackknife resampling across the genome and using Mbuti as the outgroup. A total of 353,505 SNPs from 979 samples were used in these analyses. Additional *f4*-statistics were computed when ancient samples were involved, using French as the outgroup to avoid deep attraction to Africans and only transversions (2,947–51,452 SNPs depending on the quality of samples) to avoid potential noise from ancient DNA damage patterns^28^. We used pheatmap package in R version 3.6.0 to visualize the heatmap of *f3* and *f4* profiles.

To examine the haplotype sharing between different groups, we used SHAPEIT version 4.1.3^29^ to phase the modern samples. We employed South Asian and East Asian populations as a reference panel (excluding the Kinh Vietnamese) and the recombination map from the 1000 Genomes Phase3^30^ was also used. Our analysis specifically focused on modern population data, consisting of 359,539 SNPs. For the preparation of the reference panel, we extracted individuals of East and South Asian descent, as well as the overlapping sites with our data, for each chromosome from the 1000 Genomes Phase3 data using bcftools version 1.4. The phasing accuracy of SHAPEIT4 can be improved by increasing the number of conditioning neighbors in the Positional Burrows-Wheeler Transform (PBWT) on which haplotype estimation is based^29^. We conducted phasing with the option -pbwt-depth 8 for 8 conditioning neighbors, while keeping other parameters as default. Subsequently, we employed ChromoPainter version 2^31^ on the phased dataset to initiate the investigation of haplotype sharing with sample sizes for each population were randomly down-sampled to 4 and 8. The former was used for 10 iterations of the EM (expectation maximization) process to estimate the switch rate and global mutation probability. The latter was employed for the chromosomal painting process with the estimated switch and global mutation rates. The output of this process was then used for downstream analyses. We then attempted to paint the chromosomes of each individual, with all the modern Asian samples serving as donors and recipients via the -a argument. The EM estimation yielded a switch rate of approximately 251.21 and a global mutation probability of approximately 0.00001, which were subsequently used as starting values for these parameters for all donors in the painting process. The heatmap results were generated using the pheatmap package in R.

### Admixture graph analyses

To construct the admixture graph, our initial step involved selecting backbone populations from different language families in Southeast Asia. Specifically, we used *f4*-statistics to choose representative ethnic groups that speak Austronesian, Tai-Kadai, Austroasiatic, Hmong-Mien, and Sino-Tibetan languages, which included Atayal, Dai, Cambodian, Miao, and Naxi, respectively. We employed the African Mbuti and North Indian populations (Gujarati, Brahmin Tiwari, and Lodhi) who speak Indo-European languages as outgroups. Our focus was on constructing the admixture graph for the Austroasiatic language family in Thailand. Thus, we categorized these populations according to their linguistic branches; Katuic (Bru and Soa), Monic (Mon), Palaungic (Lawa_Eastern, Lawa_Western, Palaung, Blang), and Mlabri. Our interested Khmuic-speaking people were divided into the Khamu (consist of four Khamu populations) and Lua (consist of two Lua populations together with HtinMal and HtinPray).

For modeling the admixture graph, we used a dataset of 359,539 SNPs from modern populations as the input for ADMIXTOOLS 2^32^. Initially, we computed pairwise *f2* statistics between the groups using the “extract_f2” function with specific parameters; “maxmiss = 0” (no missing SNPs to calculate), “useallsnp: NO” (no missing data to allow), and “blg = 0.05” (SNP block size set in 0.05 morgans). Then, we extracted allele frequency products from the computed *f2* blocks using “f2_from_precomp”. Next, for each scenario, we searched for the best-fitting admixture graph by running ten independent runs of “find_graphs”. From the 100 independent runs, we selected the one with the lowest score (computed based on residuals between the expected and observed *f*-statistics given the data) using “random_admixturegraph”. To confirm the fitting graph, we tested the graph with the lowest score using “qpgraph” with parameters “numstart = 100, diag = 0.0001, return_fstats = TRUE”. This allowed us to check if the absolute value of the worst-fitting Z score was below 3. Starting with no migrations (numadmix = 0), we gradually added migrations until we found a fitting graph, which we considered as the best-fitting graph for that particular scenario.

### Supplementary Information

Supplementary Figures.

Supplementary Tables.

## Data Availability

The datasets generated and analysed during the current study are available in the Zenodo repository (https://doi.org/10.5281/zenodo.8129076).
