# Supplementary material for: Genetic diversity and ancestry of the Khmuic-speaking ethnic groups in Thailand: a genome-wide perspective
Source: Sci Rep. 2023 Sep 21;13:15710. doi: 10.1038/s41598-023-43060-7 (PMC10514191; doi:10.1038/s41598-023-43060-7)

**Supplementary Figure 1** A high-resolution image of the plot depicting PC1 versus PC2 for the genome-wide SNP data of individuals from South Asia, Northeast Asia, and Southeast Asia. Each individual is colored according to their linguistic family, as indicated by the key in the right panel.

**
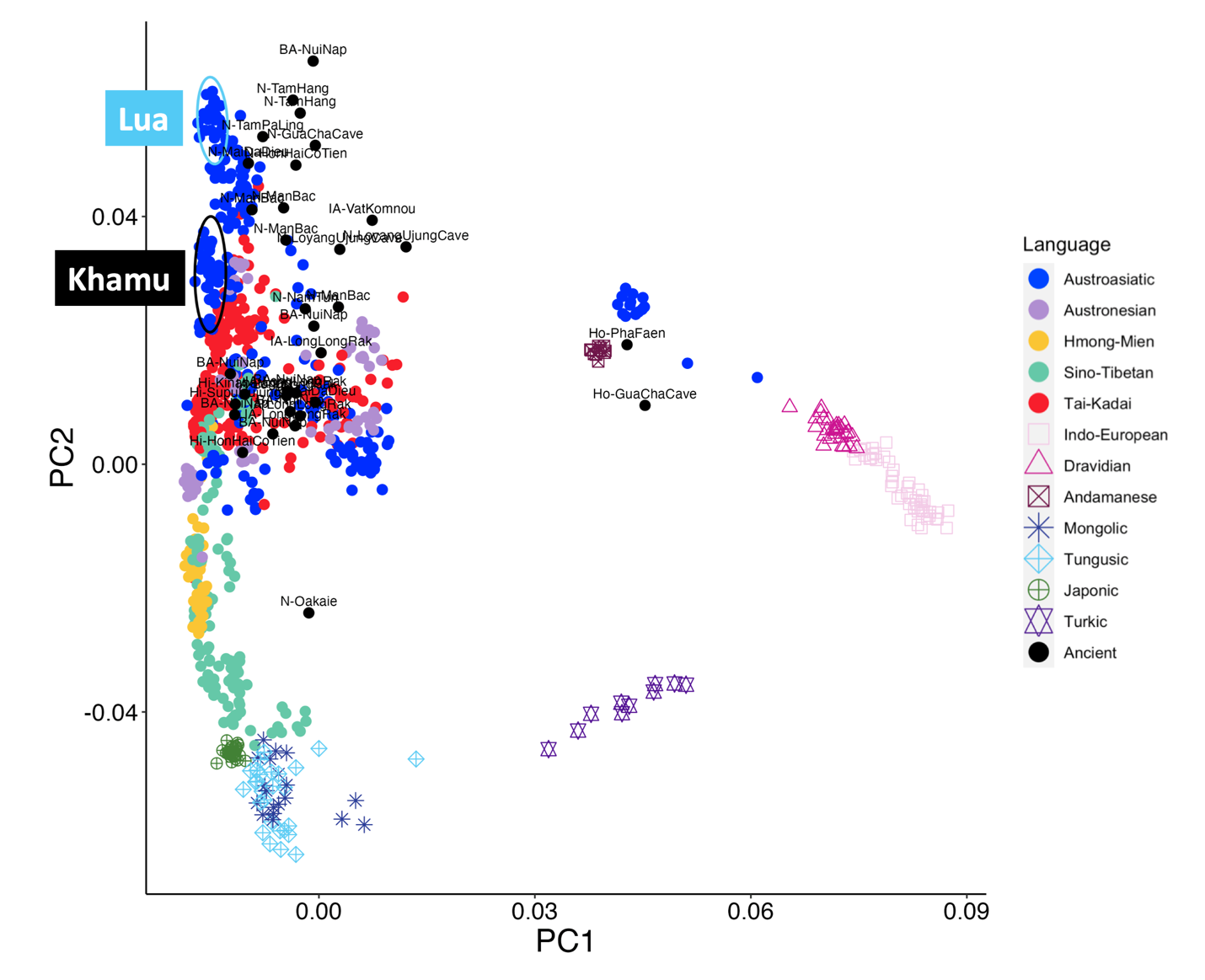
**

**Supplementary Figure 2** Cross validation errors of ADMIXTURE runs for *K*= 2 to *K* = 10, based on 100 runs for each *K* value.


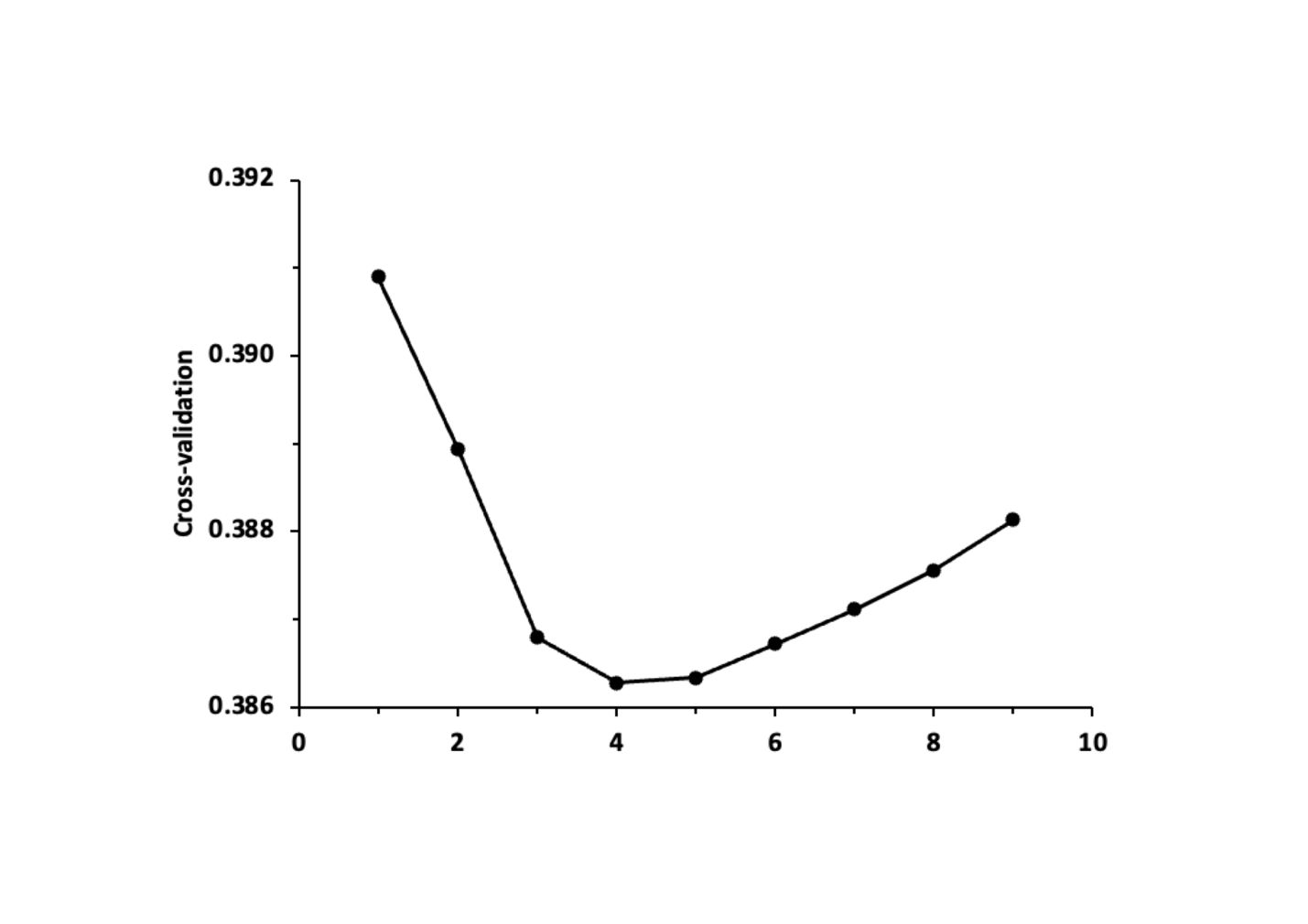


**Supplementary Figure 3** ADMIXTURE results of Southeast Asian ancient DNA samples for K values ranging from 2 to 10. Each individual is represented by a bar divided into K colored segments, indicating their estimated membership fractions in each of the K ancestry component. Populations are separated by black lines.

**
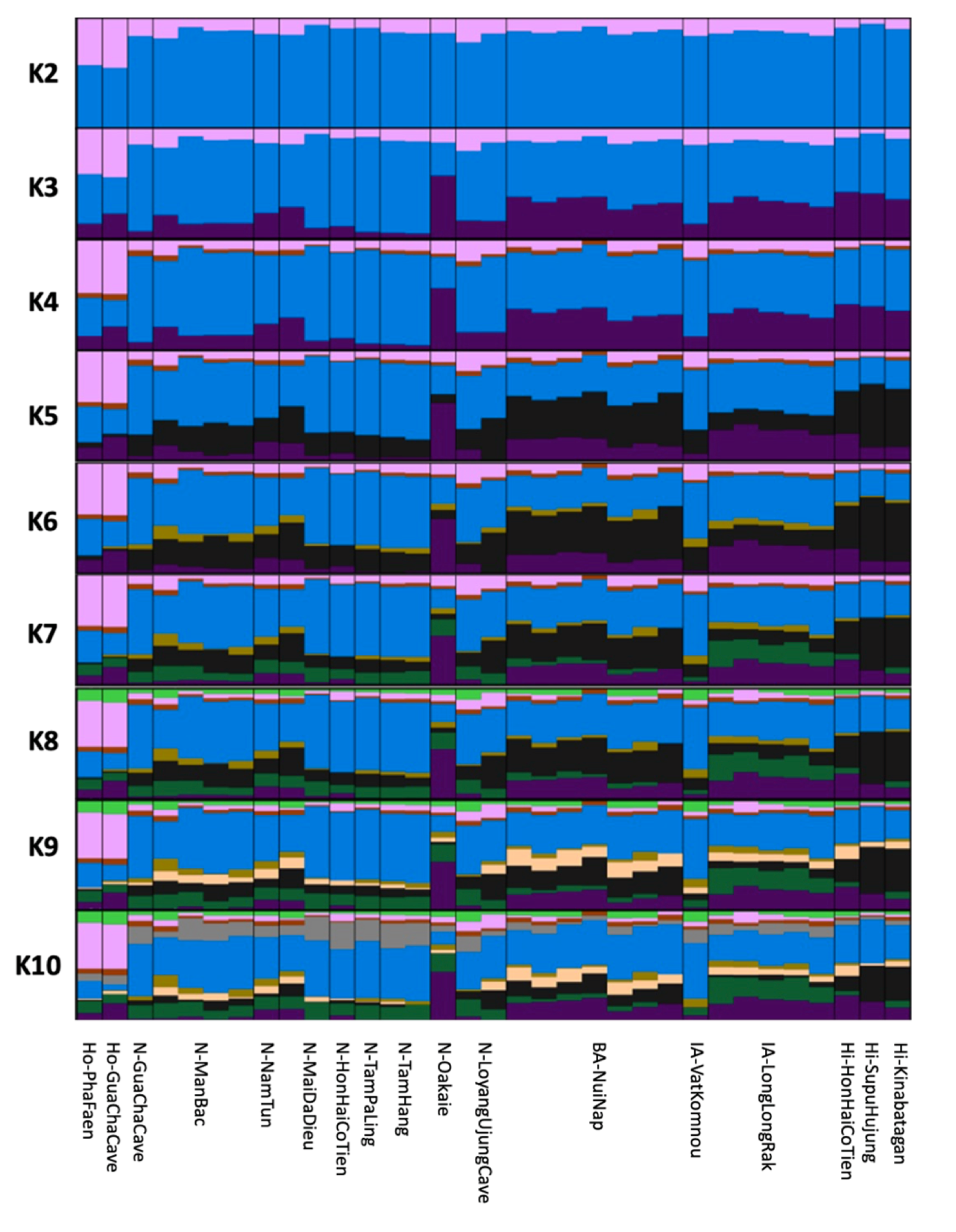
**

**Supplementary Figure 4** Heatmap of population allele sharing profiles based on f3 statistics between modern Asian populations labeled on the Y-axis and ancient samples on the X-axis. The colored bar on the right indicates the statistical values, while that on the left side indicates the linguistic family of each ethnic group.


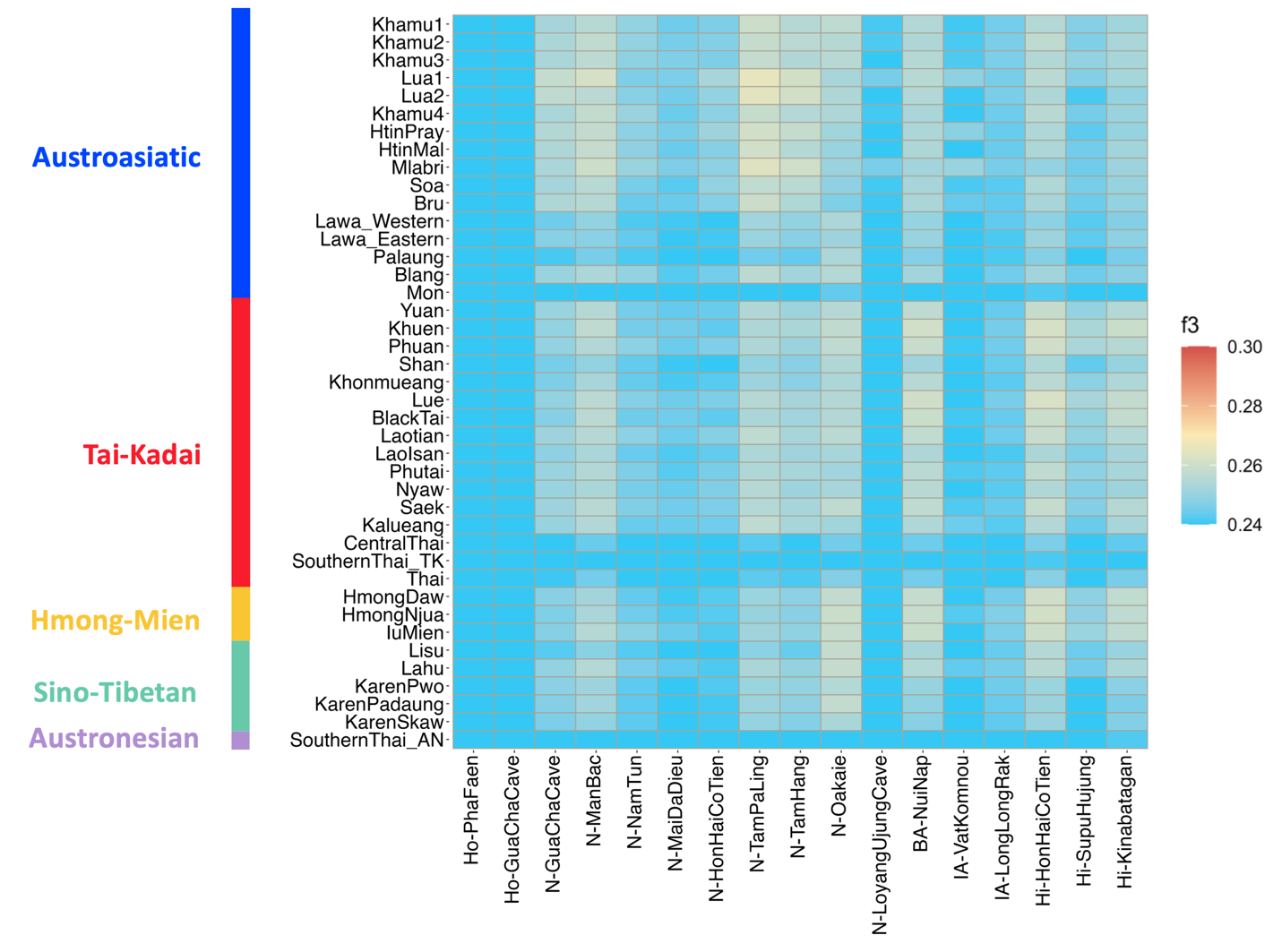


**Supplementary Figure 5** Heatmap showing population haplotype sharing profiles inferred by the ChromoPainter analyses. The colored bar on the right indicates the statistical values, while that on the left side indicates the linguistic family of each ethnic group. The Khmuic-speaking populations are emphasized with a black box in the upper-left corner.

**
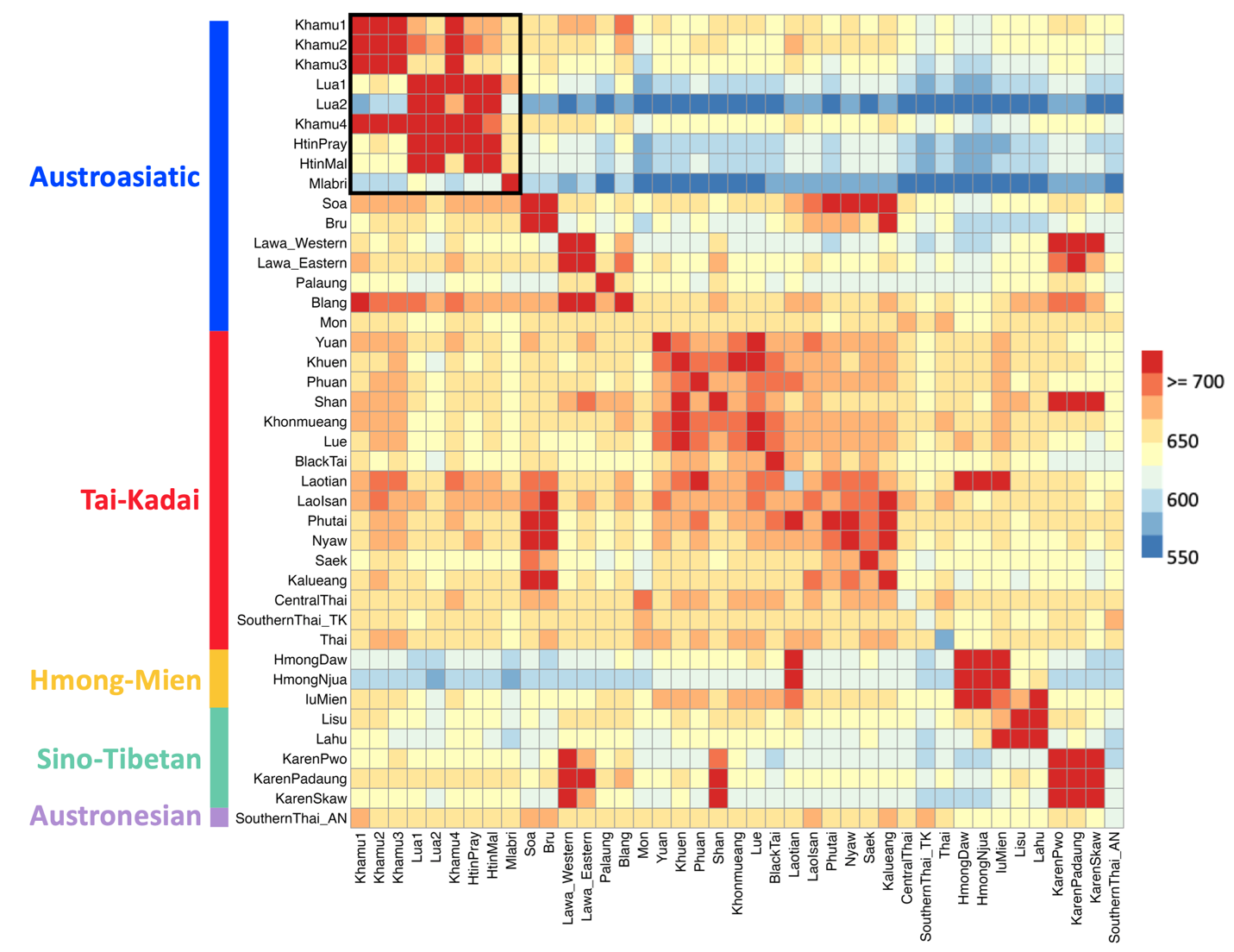
**

**Supplementary Figure 6** *f4* statistics comparing ethnic populations in Thailand labeled on the left to ancient samples on the upper grey bar. Z-scores are for *f4* (ancient sample, Han Chinese; ethnic populations, French). The vertical grey lines denote 0. The dots and error bars are colored according to language family, as indicated by the key at the bottom. Empty circles denote nonsignificant Z-scores (|Z|=<3) and solid circles denote significant Z-scores (|Z|>3).


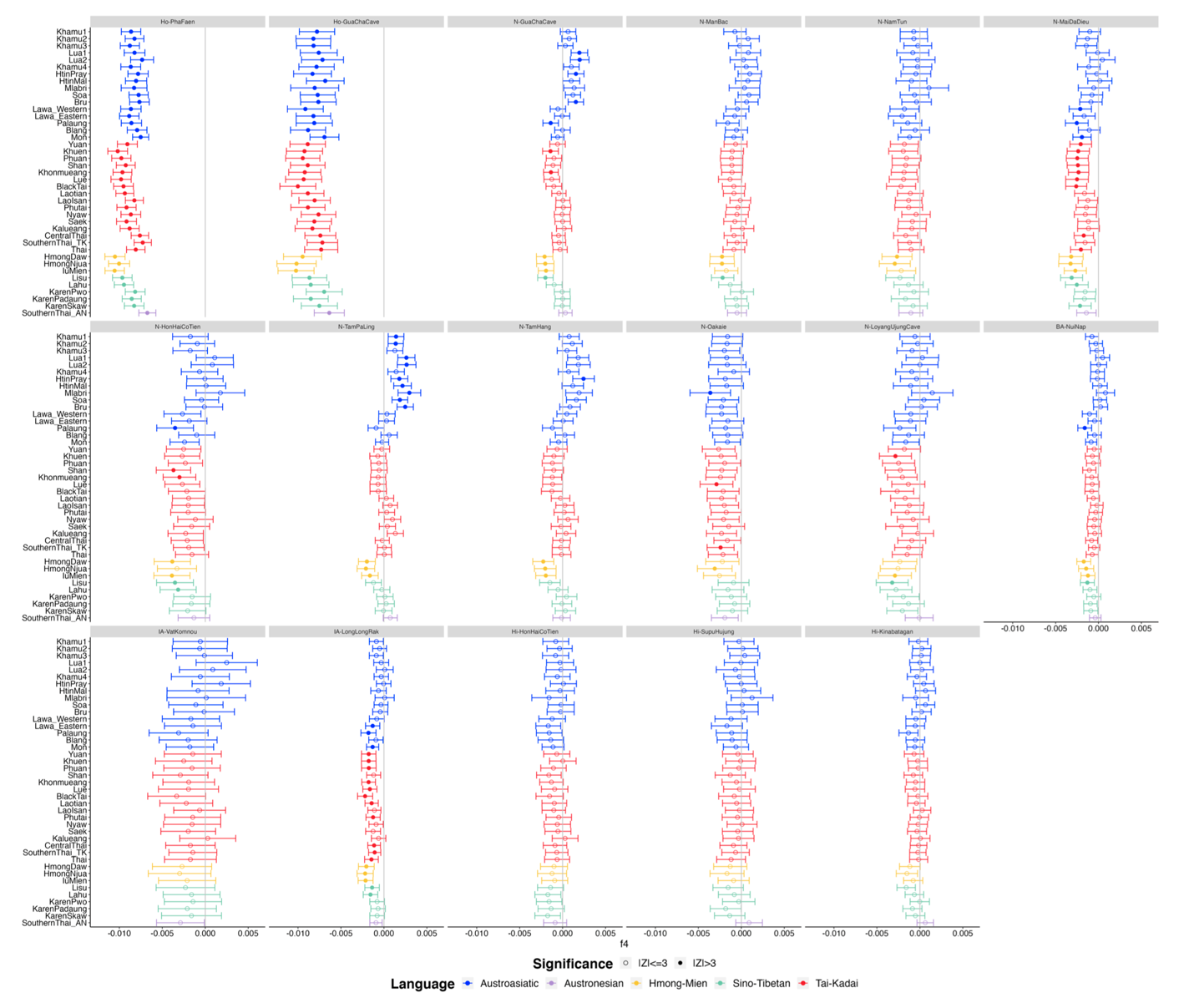


**Supplementary Figure 7** *f4* statistics comparing ethnic populations in Thailand labeled on the left to representative East Asian populations on the upper grey bar and Han Chinese. Z-scores are for *f4* (W, Han Chinese; Y, Mbuti), where W is the selected East Asian population (panel labels) and Y is the ethnic population in Thailand (label on the Y axis). The vertical grey lines denote 0. The dots and error bars are colored according to language family, as indicated by the key at the bottom. Empty circles denote nonsignificant Z-scores (|Z|=<3) and solid circles denote significant Z-scores (|Z|>3).


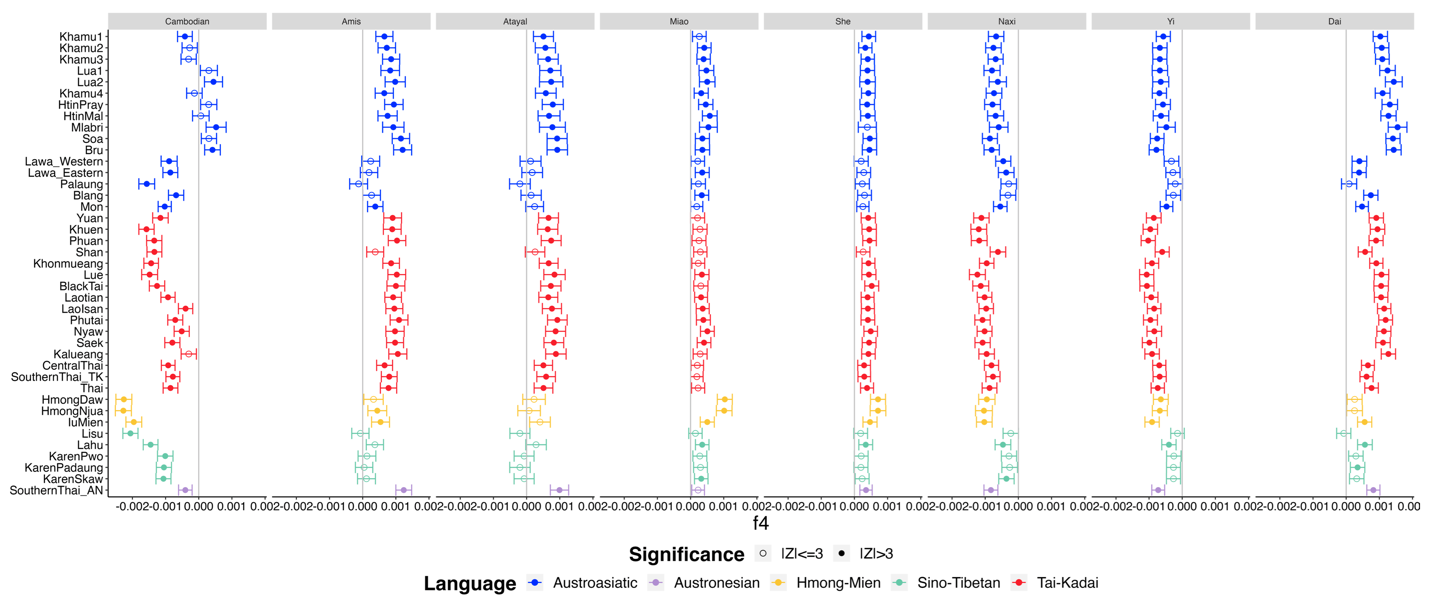

Supplement: Supplementary file 1 — Supplementary Figures. [file 41598_2023_43060_MOESM1_ESM.docx]
